# Effect of Different Colours of Light on Chosen Aspects of Metabolism of Radish Sprouts with Phosphoromic Approach

**DOI:** 10.3390/molecules29235528

**Published:** 2024-11-22

**Authors:** Anna Kafka, Jacek Lipok, Beata Żyszka-Haberecht, Dorota Wieczorek

**Affiliations:** Department of Pharmacy and Ecological Chemistry, Institute of Chemistry, University of Opole, Oleska 48, 45-052 Opole, Poland; anna.kafka@uni.opole.pl (A.K.); jacek.lipok@uni.opole.pl (J.L.)

**Keywords:** plant metabolism, abiotic stress, phosphorus, light, phosphorome

## Abstract

Among various environmental factors, light is a crucial parameter necessary for the germination of some seeds. Seed germination is an important phase in the plant life cycle, when metabolic activity is resumed and reserves are mobilized to support initial plant development. Although all nutrients are extremely important for proper physiological and biochemical development of plants, phosphorus (P) seems to play a special role, as it is an essential component of all important structural and functional substances which compose the cells of plants. We believe that transformations of the forms of phosphorus accompanying metabolic activity of germinating seeds determine the efficiency of this process, and thus the seedling’s metabolic status. Therefore, we decided to study the changes in the major phosphorus-containing substances in radish sprouts during the first phase of growth. The effect of different colours of light on the quality parameters in radish, as a model plant, during germination, was evaluated. Contents of Pi, adenylates, antioxidants, pigments, phytase activity, and ^31^P NMR phosphorus profile were investigated. Based on the results of our study, we may propose the phosphoromic approach as an important metabolic parameter determining the physiological status of the plant.

## 1. Introduction

Metabolism refers to the different chemical processes going on continuously inside living cells that allow them normal functioning. Metabolic status means readiness to take some action, in the form of a specific biochemical response, which helps to maintain the homeostasis of an organism and in consequence its survival and development. The transformations of the forms of phosphorus accompanying metabolic activity in the living being determine its metabolic efficiency. The management of various forms of phosphorus is one of the key processes in this area. Plant phosphorus requirements are greatest in the early stages of development—germination and seedling formation—so plant seeds accumulate and store the phosphorus required for the initial growth of seedlings. The “phosphorome” represents a novel approach to the study of phosphorus metabolism in plants [1,2]. From a metabolic point of view, ATP seems to be the most important phosphorus compound in living cells, which regulates internal homeostasis, metabolic activity and susceptibility to physiological stress. In turn, the proportional concentrations of the nucleotides ATP, ADP and AMP reflect the metabolic activity of an organism that is suppressed by environmental factors. The ratio of particular nucleotides may be expressed as adenylate energy charge (AEC). AEC values indicate the switch between anabolic and catabolic pathways, which allows for deeper analysis of the metabolic state of the plant’s living cells [3,4]. Cellular responses to various environmental conditions also include changes of other chemical markers of metabolism such as the content of photosynthetic pigments, antioxidant compounds and total protein. These indicators provide insights into the plant’s response to stress and facilitate the understanding of the underlying mechanisms involved.

Light, as the original source of energy for photoautotrophs, is one of the most important factors affecting plant growth and phytochemical concentrations in cells and tissues [5]. Different characteristics of light, such as spectral composition (wavelengths), intensity, or duration, can influence the growth and development of plants. For plant cultivation, different conventional light sources such as metal halide or high-pressure sodium lamps can be used. In recent years, light-emitting diodes (LEDs) have been gaining in importance. LED systems have many advantages over other light sources. Among others, they are characterized by durability, small size, long operating lifetimes, low energy consumption, and wavelength specificity [6]. Regardless of the type of light source, it has been shown that light may stimulate or inhibit the germination of some seeds. Depending on the plant species, some seeds germinate better in either light or darkness, while others germinate regardless of the presence or absence of light [7]. To receive light, plants have evolved photoreceptors, such as red light-sensitive phytochromes, blue light-sensitive phototropins, and cryptochromes, which regulate several physiological responses, including organogenesis and metabolite synthesis [8]. The influence of light on plant physiology varies between species, often resulting in significant variations in biomass yield or phytochemical composition [8]. Red and blue light seem to have the greatest impact on plant growth and metabolism because they provide the primary energy for photosynthesis in plants [9]. However, yellow, green, or ultraviolet (UV) lights also have effects in this regard [10]. Under blue light, an increase in the biosynthesis of compounds such as carotenoids, chlorophylls, and polyphenols (e.g., chlorogenic acid) has been observed in plants and microgreens [11]. Exposure of some plants to red light also enhances the content of phenolic compounds, while a combination of red and blue light leads to the accumulation of nitrogen in the leaves [12]. Yellow and green light influences the content of isoflavones in legume sprouts [13] and UV light can lead to the accumulation of, e.g., flavonoids, glucosinolates, or carotenoids in plant cells [14,15].

Germination (sprouting) is a bioprocess that enhances the nutritional value of seeds. The processes taking place during this stage lead to the activation of different types of enzymes which break down stored substances such as proteins, carbohydrates and lipids into simpler and more easily absorbed forms [16]. The conditions that prevail during the sprouting stage are of great consequence, as they frequently serve to determine the ultimate composition of the sprouted seeds. The environmental factors that are responsible for ongoing changes in seeds, including radish, during the sprouting process include: deficiency (drought) or excess water, lighting (intensity, wavelength, photoperiod), temperature, deficiency or excess of nutrients in the macro- and microelements, salinity, presence of toxic metals or xenobiotics [17]. These factors, when outside their normal ranges, have negative biochemical and physiological consequences for plants. Although plants tolerate some deviation from homeostatic equilibrium, their optimal functioning is achieved within a narrow range of changes in the factors that characterise a given ecosystem [18]. Any more significant change in the conditions of the plant’s environment constitutes a stress factor. Thus, an environmental stressor is any external factor that has an adverse effect on the plant, leading to physiological changes that result in a new state of homeostasis [19,20]. However, continuous and prolonged exposure to stressors contributes to a deterioration in the physiological state of the plant, which can eventually lead to irreversible changes and, in extreme cases, to the death of the organism [21,22]. The extent of physiological stress can be quantified by assessing differences in selected parameters—biochemical markers in plants developing under optimal or unfavourable growth conditions.

Considering that light stress is a convenient factor to study abiotic stress due to its non-invasiveness and high evolutionary conservation of coping mechanisms, our study aimed to study the effect of various colours of light on the quality parameters in a model plant—radish sprouts—during germination. Phytase activity, germination profile of the seeds, and the content of (i) inorganic phosphorus (Pi), (ii) proteins, (iii) adenine nucleotides (AMP, ADP, ATP), (iv) photosynthetic pigments (total chlorophyll and carotenoids), (v) phenolic compounds, (vi) flavonoids, (vii) antioxidant compounds and their antioxidant capacity were determined. ^31^P NMR phosphorus profiles and energy status (AEC) constitute another interesting aspect presented in this paper.

## 2. Results

Seed germination represents a pivotal phase in the life cycle of plants. During the course of evolution, plants have developed appropriate mechanisms to detect and respond to environmental conditions that are conducive to germination. The alterations occurring within the cells are specific signals that facilitate the plant’s capacity to adapt to prevailing environmental conditions. The plant’s demand for phosphorus is greatest during the early stages of development, specifically during germination and seedling formation. This is due to the fact that metabolic processes are most dynamic during this period [23]. The only source of phosphorus available during the initial stages of seed germination is that which is stored in various organic compounds [2]. For this reason, phosphorus profiles in radish seeds were first determined on the basis of extractable inorganic and organic forms of phosphorus. Quantitative (Pi content) and qualitative (phosphorus profiles) phosphoromic analyses of radish seeds were carried out by measuring the contents of the relevant forms of phosphorus in seed extracts. The extracts were obtained by a sonication process (3 × 30 min using 35% (*v*/*v*) HClO_4_ solution as solvent. Control cultures, in which radish sprouts developed under optimal growth conditions, were run in parallel for each lighting system tested. Furthermore, in order to facilitate the determination of the chosen abiotic stressor, light stress, on the plant condition, the results are partially presented in the paper in relation to the reference system. The values obtained were normalised, taking the control as 100%.

### 2.1. Influence of Different Colours of Light on Seed Germination and Seedling Growth of Radish Sprouts

**Figure 1:**
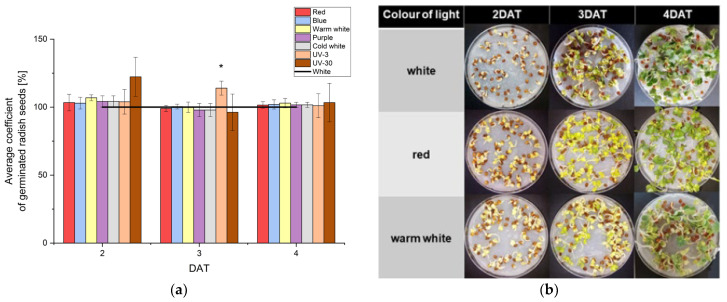
The effect of different colours of light on seed germination of radish sprouts. (**a**) The percentage of seed germination after treatment with different colours of light: white, red, blue, warm white, purple, and cold white. The values of the appropriate control cultures (white) were taken as 100%, “*” indicates statistically significant differences (*p* ≤ 0.05) in relation to the control; (**b**) the morphology of selected germinated radish seeds included the last phase of germination (2 DAT), when the appearance of the embryonic root was noticed, until the end of culture (4 DAT).

Assessment of the germination ability of radish seeds is one of the basic tests used to determine not only the quality of the seeds, but also the influence of external conditions on the germination process. In the case of radish (*Raphanus sativus* L.), the seed test was carried out on filter paper (as recommended by ISTA, 2018) [24], which means that the seeds were directly exposed to light in the phytotron where they were tested. After 24 h of dark germination (1 DAT, day after treatment), the average percentage of radish sprout germination (GP) was about 64.8 ± 6.7%, and in the following days reached an average level of 92.4 ± 2.2%, 95.1 ± 0.8% and 95.9 ± 1.4%, respectively. Figure 1a summarizes the percentage of radish seed germination after treatment with different colours of light as characterised by the ratios of the GP of the appropriate experimental cultures to the GP of the control expressed as percentage values. The figure takes into account the second day of germination (2 DAT), when the appearance of the embryonic root was clearly noticed, until the end of cultivation (4 DAT). Analysing the results, it can be concluded that more germinated seeds were recorded only for UV-30 at 2 DAT and UV-3 at 3 DAT, with differences of 22% and 14%, respectively, relative to the control. However, these differences were statistically significant only for UV-3. All seeds, regardless of the lighting used, showed clear signs of germination with the lifting of the cotyledons and the elongation of the roots. However, the radicle emerged through the seed coat slower and was noticeably more yellow in the red and yellow light-treated seeds at 2 DAT (Figure 1b). The radish seeds germinated under blue, purple, UV-3 and UV-30 lights obtained at 3 DAT showed a delay in formation of the cotyledon. All radish sprouts at 4 DAT have clear leaves and roots.

### 2.2. Changes in Pi Content, Phytase Activity and Protein Content in Radish Sprouts

**Figure 2:**
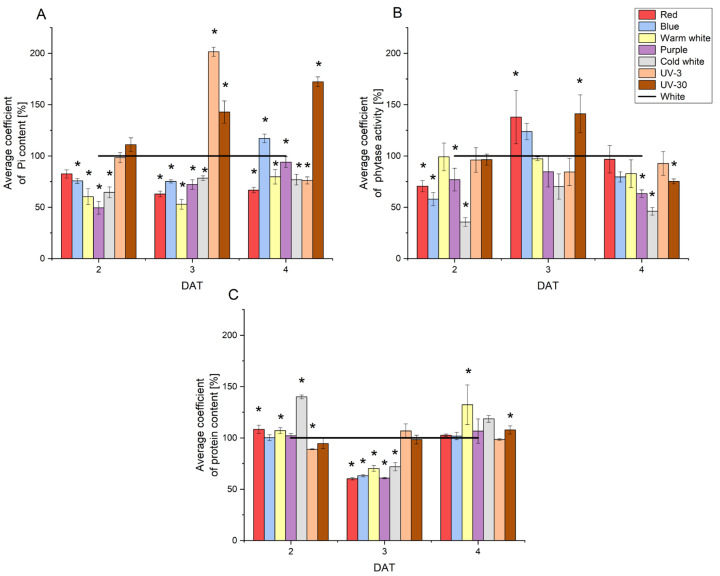
The average coefficient of Pi content (**A**), phytase activity (**B**) and protein content (**C**) in radish sprouts developing under tested light conditions. The corresponding control values were taken as 100%, ‘*’ indicates statistically significant differences (*p* ≤ 0.05) compared to the control (white).

In order to determine the effect of the tested lighting variants on selected aspects of metabolism, changes in inorganic phosphorus, protein content and phytase activity in samples of radish sprouts were analysed. The Pi content increased during germination and the average value on individual days of development was 2.45, 3.48 and 4.64 mg/g DW (2 DAT, 3 DAT and 4 DAT, respectively). The lowest level (1.35 ± 0.17 mg/g DW) was recorded at 2 DAT under purple light, while the highest (6.75 ± 0.24 mg/g DW) was determined under blue light on the last day of the experiment. The phytase activity increased during the experiment, similar to the Pi content. The average activities in the following days were 0.67, 1.15, and 1.78 U/g DW, respectively. The highest phytase activity was noted for red light on 4 DAT (3.07 ± 0.43 U/g DW), and the lowest for UV-3 light on 2 DAT (0.30 ± 0.04 U/g DW). The dynamics of the increase in phytase activity in the following days were lower in the case of UV-3 and UV-30, compared to the other light conditions tested. The average of protein content in radish sprout extracts ranged from 9.10 to 9.53 mg/g DW. The lowest value was determined for red (5.34 ± 0.10 mg/g DW) and purple (5.04 ± 0.06 mg/g DW) light at 3 DAT, while the highest (14.86 ± 0.12 mg/g DW) was recorded for UV-3 at 4 DAT. Protein content for control conditions increased from 5.52 ± 0.11 mg/g DW at 2 DAT to 8.87 ± 1.09 mg/g DW at 3 DAT, and then decreased to 5.62 mg/g at 4 DAT. The average coefficient of content of inorganic phosphorus, protein and enzymatic activity of phytase in radish samples growing in various lighting variants is shown in Figure 2.

Significant differences (*p* ≤ 0.05) in Pi content compared to the control were noted at 2, 3, and 4 DAT for all tested lighting variants. The exceptions were red light, UV-3, and UV-30 at 2 DAT. Pi content was significantly higher at 3 DAT (200% of control) and 4 DAT (172% of control) for UV-3 and UV-30, respectively. Meanwhile, significantly lower Pi content (50%, 60%, and 53% of control) was noted for purple and warm white light at 2 DAT and warm white at 3 DAT, respectively. The least impact on phytase activity was observed for warm white and UV-3 lights. Application of UV-30 resulted in a notable elevation in phytase activity at 3 DAT (140% of the control), followed by a swift and substantial decline at 4 DAT (75% of the control). Additionally, statistically significant differences were observed for red (2 and 3 DAT), blue (2 DAT), purple and cold white lights (2 and 4 DAT). The application of purple and cold white lights resulted in a reduction of phytase activity from 2 to 4 DAT. Additionally, the utilisation of red and blue light during the initial stages of radish development led to a reduction in phytase activity, with a 34% and 42% decrease observed at 2 DAT in comparison to the control conditions, respectively. Statistically significant differences in protein content compared to the control were noted for red, warm white and cold white lights at 2 DAT and warm white and UV-30 lights at 4 DAT. Protein content for all light conditions (except UV) at 3 DAT was 1.5-fold lower than for the control.

### 2.3. Phosphorus Speciation of Radish Sprouts Seeds by ^31^P NMR

The quantitative evaluation of the specific groups of phosphorus compounds was based on the chemical shift values and the area of the corresponding peaks. The P forms present in neutralised extracts from radish sprouts and the relative percentage content of the individual P forms (P1—polyphosphates/diphosphates [(−15)−(−3.5) ppm]; P2—phosphodiesters [(−1)−1.5 ppm]; P3—phytates/orthophosphates [1.5−3.7 ppm]; P4—other monoesters [3.7−6 ppm]) per total NMR area are shown in Table 1. In order to determine the effect of light on the development of radish sprouts, the table also gives the relative amounts (%) of the different forms of phosphorus determined in extracts of radish sprouts developing under optimum growth conditions.

**Figure 3:**
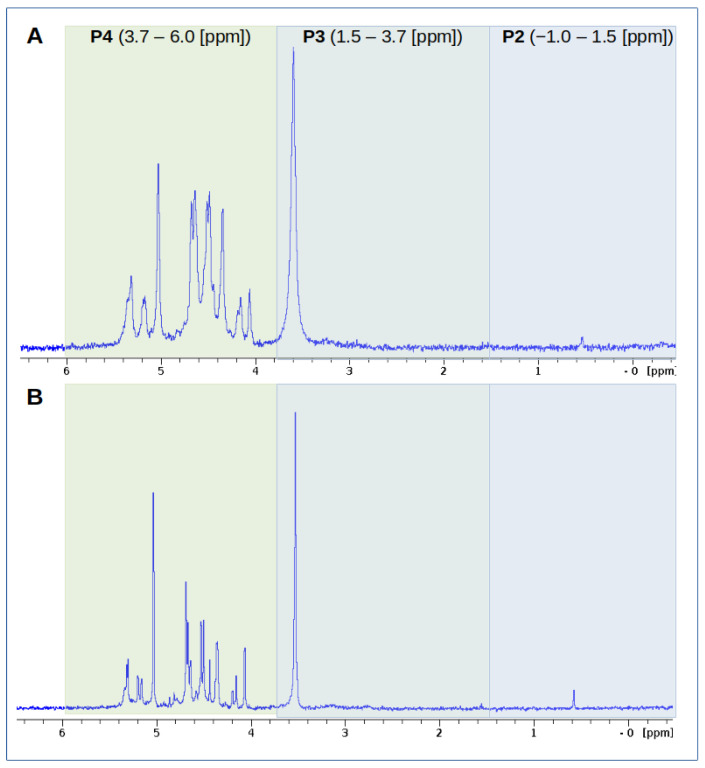
Comparison of ^31^P NMR spectra of radish sprouts (4 DAT) developing under white (control) conditions (**A**) and under cold white light (**B**). Measurements were carried out on samples at neutral pH (pH = 7); the coloured boxes surround the signals in the spectrum belonging to the different forms of P: P2—phosphodiesters; P3—phytates/orthophosphates; P4—other monoesters [3.7–6 ppm]).

The results obtained reflect the effect of different variants of light on the relative quantitative contribution of the forms of phosphorus in radish sprouts. As can be seen from the data presented in Table 1, the content of the different forms of phosphorus in radish during the germination phase varied over the duration of the experiment. On 2 DAT, phosphorus is present in the form of other monoesters (P4), the content of which in the neutralised extracts ranged from 48.6 for UV-3 to 67.2 for cold white light. The exception was UV-30, where the predominant P form was phytate and orthophosphate (P3). Figure 3 summarises examples of ^31^P NMR spectra obtained for radish sprout extracts developing for 4 days under control and cold white light conditions. The greatest differences in signal shape can be seen in the P4 interval, corresponding to phosphomonoesters. The P1 forms of phosphorus were not indicated in all tested samples.

### 2.4. Alterations in Adenylates Content and Adenylate Energy Charge Values in Radish Sprouts Cultivated Under Disparate Light Conditions

Adenylates (adenine nucleotides)—AMP, ADP, and ATP—undergo dynamic changes as a consequence of metabolic processes. Alterations in their content and their mutual quantitative ratio can provide information about the energetics of metabolic processes occurring in plants.

**Figure 4:**
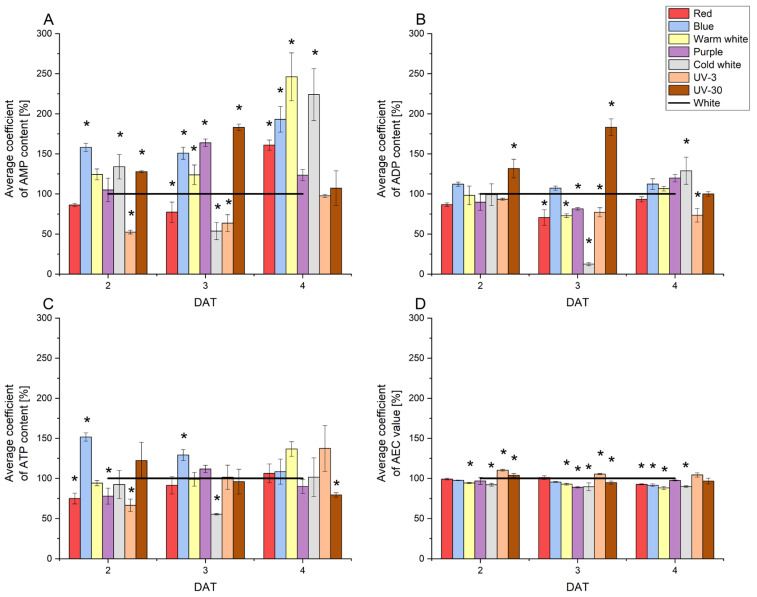
The average coefficient of adenylates: AMP (A), ADP (B), and ATP (C) and AEC parameter (D) in radish sprouts cultivated under disparate light conditions. The corresponding control values were taken as 100%, ‘*’ indicates statistically significant differences (*p* ≤ 0.05) compared to the control (white).

Among all the determined forms of adenine nucleotides, ADP was found to be the most abundant. The average content of this nucleotide in radish sprouts in the subsequent days of growth was 466.48, 552.06, and 748.91 µg/g DW (2 DAT, 3 DAT, and 4 DAT, respectively). The lowest content was recorded for cold white light at 3 DAT, while the highest was at 4 DAT. The application of this type of light resulted in notable alterations in the ADP content during the following growth days, with a decrease from 513.00 ± 69.83 µg/g DW at 2 DAT to 101.89 ± 15.45 µg/g DW at 3 DAT, followed by an increase to reach 1052.16 ± 138.47 µg/g DW at 4 DAT. The lowest concentration of AMP was observed in UV-3 light at 3 DAT (81.02 ± 13.21 µg/g DW), while the highest concentration was determined in blue light at 2 DAT (254 ± 8.24 µg/g DW). A one-day decrease in AMP content was noted in plants subjected to illumination with red, warm white, cold white or UV-3 light at 3 DAT. In the case of blue light, the amount of AMP decreased during cultivation, similarly to ATP, the content of which decreased from 195.33 ± 6.57 µg/g DW at 2 DAT to 89.09 ± 12.88 µg/g DW at 4 DAT. A similar dependence was noted in the case of UV-30 light, for which the ATP content decreased from 75.64 ± 14.10 to 39.51 ± 1.35 µg/g DW. In radish sprouts cultivated under red and UV-3 light, the ATP content remained relatively constant. In contrast, under warm white and cold white light, a decrease in ATP content was observed at 3 DAT. Figure 4 illustrates the average coefficient of content of adenine nucleotide and the AEC parameter in radish sprouts cultivated under diverse lighting conditions.

Blue light increased the AMP content by an average of 70% compared to the control. In the case of warm white light, this increase was almost 1.5-fold higher at 2 and 3 DAT and more than twice as high at 4 DAT. In the case of cold white light, the AMP content was 1.5-fold higher at 2 DAT compared to the control conditions, then it dropped dramatically at 3 DAT, and at 4 DAT it was more than twice as high as compared to the control. A similar effect was noted for purple light, which at 3 DAT caused a significant increase in the AMP content by 100% compared to the control. A much smaller effect of light on the content of adenine nucleotides was observed for ADP and ATP. For each of the lighting conditions, the ADP content on 3 DAT was lower compared to the control, except for blue and UV-30 light. It is also worth noting that the ratio of ADP content for UV-30 light to ADP content for the control in subsequent days of radish sprout development is the same as the ratio of AMP content. As with the changes in AMP content, blue light significantly increased ATP content compared to the control at 2 and 3 DAT. On the other hand, the effect of cold white light led to a significant decrease in the content of all adenine nucleotides at 3 DAT. Statistically significant differences in ATP content compared to the control were also noted for red, purple, and UV-3 light at 2 DAT and for UV-30 light at 4 DAT.

A more precise determination of the effect of light treatment on the metabolism of radish sprouts was made possible by utilising the adenylate energy charge (AEC) value. The AEC parameter values ranged from 0.40 (for cold white light at 2 DAT) to 0.50 (for UV-3 light at 4 DAT). A statistically significant effect on the energy status of radish sprouts was determined for warm white and cold white light on each day of cultivation. Additionally, a statistically significant effect was observed for purple light at 3 DAT, UV-3 and UV-30 light at 2 and 3 DAT, as well as for red and blue light at 4 DAT. Concurrently, the AEC parameter value on individual days remained at a relatively constant level (SD ≤ 0.06) for all experimental variants.

### 2.5. The Content of Photosynthetic Pigments in Radish Sprouts in Regard to Different Light Treatments

**Figure 5:**
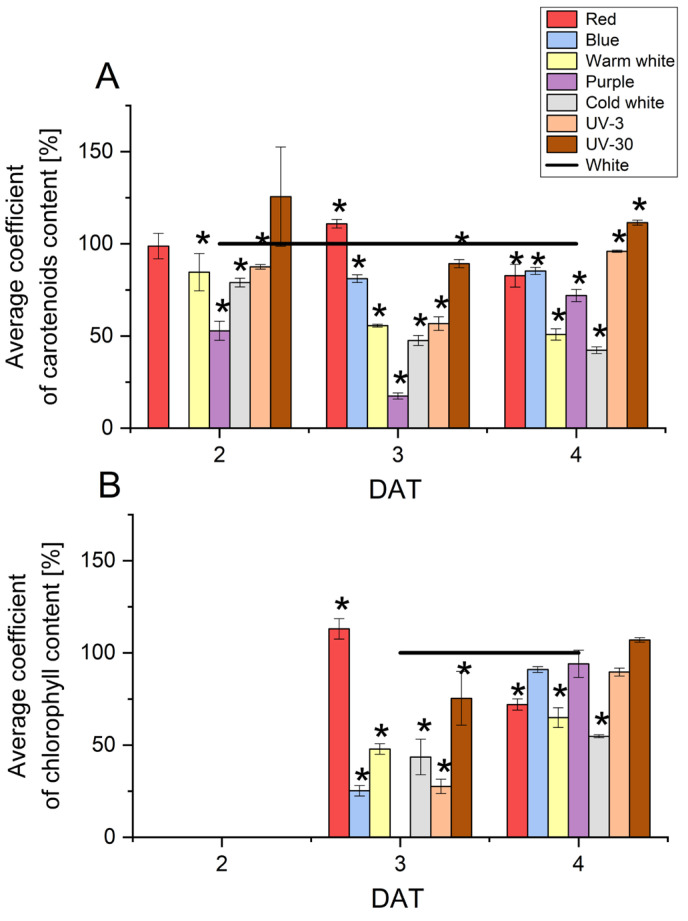
Average coefficient of carotenoids (**A**) and total chlorophyll (**B**) content in radish sprouts developing under the light conditions tested. Values for the respective control cultures were taken as 100% in each case, ‘*’ denotes statistically significant differences (*p* ≤ 0.05) with respect to the control (white).

The process of seed germination and initial sprout growth is a short phase in the development of plants without photosynthesis and is therefore a critical phase in the growth of plants, which are then particularly vulnerable to external stresses. Tracking the activation of the photosynthetic process at such an early stage of plant development is possible, among other things, by determining the content of photosynthetic pigments—compounds responsible for absorbing light energy. Figure 5 shows the changes in mean carotenoid and total chlorophyll content over the duration of the experiment.

The average content of naturally occurring carotenoids in radish seeds ranged from 0.02 to 0.16 mg/g DW and increased over time. The highest content (0.26 mg/g DW) was determined at 4 DAT for red and blue light, while the lowest content (0.01 mg/g DW) was determined for purple light at 2 DAT. Furthermore, in the early days of culture (2 DAT) for blue light, the carotenoid content was below the level of quantification. The greatest negative effect of light on carotenoid content was observed for purple light and cold white light, where the differences with respect to control conditions were smaller: 80% at 3 DAT for purple light and 60% at 2 and 4 DAT for cold white light.

Total chlorophyll content was only determinable from 3 DAT (except for purple light), and the average content ranged from 0.10–0.64 mg/g DW. Although red light did not affect the germination rate, it contributed to a 13% increase in chlorophyll content at 3 DAT. In contrast, a negative effect of illumination on chlorophyll content was recorded for blue and UV-3 light at 3 DAT (by an average of 75%), and warm white and cold white light at 3 and 4 DAT (by about 55% and about 40%, respectively).

### 2.6. Changes in Antioxidant Capacities and Content of Antioxidant, Phenolic Compounds and Flavonoids in Radish Sprouts Developing Under Various Light Conditions

The activation of plant defence systems in response to environmental stress is accompanied by the induced synthesis of secondary metabolites exhibiting antioxidant activity [25,26].

**Figure 6:**
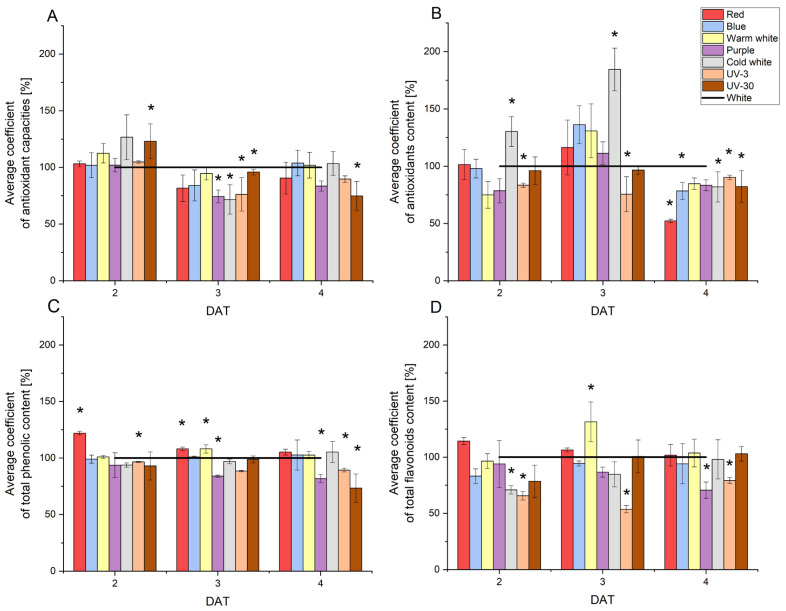
Average coefficient of antioxidant activity (**A**) and content of antioxidants (**B**), phenolic compounds (**C**), and flavonoids (**D**) in radish sprouts developing under the tested light conditions. Values for the respective control cultures were taken as 100% in each case, ‘*’ denotes statistically significant differences (*p* ≤ 0.05) with respect to the control (white).

The average antioxidant activity (RSA) of radish sprouts was observed to range from 62 to 73%. The lowest RSA value was recorded for sprouts developing in cold white light (52.7%) and purple light (54.7%) at 3 DAT, while the highest was observed in blue light (79.7%) and cold white light (79.3%) at 4 DAT. At the initial stage of growth (2 DAT), radish sprouts exposed to warm white and cold white light exhibited a higher antioxidant capacity (by 12% and 27%, respectively) than the control (Figure 6). Significant effects of lighting on antioxidant activity were observed for UV-30 light, which increased the RSA value by 23% at 2 DAT and decreased it by 5% at 3 DAT and by 25% at 4 DAT compared to white light. The application of purple, cold white and UV-3 light resulted in a reduction in antioxidant activity at 3 DAT, with an average decrease of 26% in comparison to the control. A detailed examination of the presented results reveals a notable increase in antioxidant content following the application of cold white light at both 2 and 3 DAT. Specifically, a 30% and 85% enhancement were observed at these time points, respectively. Conversely, at 4 DAT, a notable decline in antioxidant content was observed for red light (by 48%), blue light (by 22%), cold white light (by 18%), and UV-30 (by 18%). The average content of phenolic compounds increased during radish development, reaching a range of 11.0–13.5 mg GAE/g DW. The lowest recorded content (8.69 mg GAE/g DW) was observed for UV-30 at 2 DAT, while the highest (16.1 mg GAE/g DW) was noted for red and cold white light at 4 DAT. The application of red light resulted in a notable elevation in the concentration of phenolic compounds, amounting to 22% (2 DAT) and 8% (3 DAT) increases, respectively. A comparable effect was observed for warm white light at 3 DAT (an increase in content by 8%). Conversely, a statistically significant decrease in the content of phenolic compounds was noted for purple light at 4 DAT (by 18%), UV-3 at 2 DAT (by 3%) and 4 DAT (by 11%), and UV-30 at 4 DAT (by 27%). The average flavonoid content in radish sprouts was found to range from 4.1 to 4.8 mg Q/g DW. The lowest content (2.7 mg Q/g DW) was observed at 3 DAT for UV-3, while the highest (5.5 mg Q/g DW) was recorded at 4 DAT for red light. An increase in flavonoid content was observed in radish plants exposed to red light at 2 DAT and warm white light at 3 DAT. However, only the increase in flavonoid content observed in response to warm white light was statistically significant. Conversely, significantly lower flavonoid content was observed for purple light at 4 DAT (by 30%), cold white light at 2 DAT (by 30%), and UV-3 at 2 DAT (by 34%), 3 DAT (by 46%), and 4 DAT (by 20%).

## 3. Discussion

Metabolism involves continuous chemical processes in living organisms that ensure normal functioning and homeostasis, crucial for survival and development. One of the most important elements determining the metabolic efficiency of each organism is phosphorus. In the case of plants, the efficiency of transformations of phosphorus compounds determines the organism’s readiness to carry out specific biochemical reactions that enable its development and maintain homeostasis, including critical growth stages like germination. Research in this area is made feasible by implementing a novel approach in metabolomics, namely phosphoromic research. The phosphorus compounds which play a role as a source of energy for use and storage at the cellular level are adenylate nucleotides. The ratios of ATP, ADP, and AMP reflect metabolic activity, influenced by environmental factors, and are expressed as adenylate energy charge (AEC), indicating whether a plant is in anabolic or catabolic mode.

Light, as the original source of energy for photoautotrophs, is one of the most important factors affecting plant growth and phytochemical concentrations in cells and tissues [27]. Research indicates that the long-term growth of different radish varieties is dependent on the optimal light spectrum, affecting, among other things, morphology, growth rate, photosynthesis, and antioxidant capacity [28]. When this agent is outside its normal range, it usually has negative biochemical and physiological consequences for plants. Although plants tolerate some deviation from homeostatic equilibrium, their optimal functioning is achieved within a narrow range of changes in the factors that characterise a given ecosystem. Considering that light stress is a convenient model to study abiotic stress due to its non-invasiveness and high evolutionary conservation of coping mechanisms, our study aimed to study the effect of white, cold white, warm white, red, blue, and purple light on quality parameters in radish sprouts during germination. Phytase activity, germination profile of the seeds, and the content of (i) inorganic phosphorus (Pi), (ii) proteins, (iii) adenine nucleotides (AMP, ADP, ATP), (iv) photosynthetic pigments (total chlorophyll and carotenoids), (v) phenolic compounds, (vi) flavonoids, (vii) antioxidant compounds and their antioxidant capacity were determined. ^31^P NMR phosphorus profiles and energy status (AEC) constitute another interesting aspect presented in this paper.

Cellular responses to stress also include changes in metabolism and the cell cycle [29]. The noticeable differences in plant morphology that occur as a result of physiological stress are preceded by changes in the concentrations of various chemical markers of metabolism. These indicators provide insights into the plant’s response to stress and facilitate understanding of the underlying mechanisms involved. Adequate phosphorus contents not only increase the percentage of germinated seeds, but also reduce germination and sprout growth time. Based on the results presented, it can be concluded that light has some effect on radish seed germination. This may be related to the synthesis and conversion of gibberellins, germination-promoting hormones, through the activation of phytochromes (Pfr) [30,31,32,33].

Changes in Pi content during seed germination result from differences in metabolic processes. Higher content of Pi in radish sprouts treated with UV-3 (3 DAT) and UV-30 (4 DAT) indicates the release of Pi from phosphomonoesters as a result of hydrolysis of, for example, sugar derivatives. Plant “phosphorome” studies can be enriched by assessing phytase activity as a new metabolic trait in determining the condition of plants developing under stress conditions [2,34]. One of the most important reactions occurring in developing sprouts is the hydrolysis of ester bonds in phytate molecules, as a result of which phosphorus stored in the embryo is released. These reactions are catalysed by phytases. The studies conducted have shown that red, blue, purple, and cold white lights cause a negative impact on the enzymatic activity of phytases during radish seed germination, which may indirectly indicate a disturbance in the catalytic efficiency of the enzymes tested. Unfortunately, the current lack of information does not allow for a detailed determination of the effect of the tested lighting conditions on the catalytic activity and substrate specificity of phytases in radish sprouts. The type and activity of phytases depends on the species and even the variety of the plant [35,36]. Most phytases present in plant tissues are classified as histidine acid phosphatases (HAP), which exhibit broad substrate specificity and, in addition to dephosphorylating phytic acid, can catalyse the hydrolysis of other phosphate esters [2,35,36,37]. The protein concentration in plant tissues is dependent upon the equilibrium between the proteolysis of accumulated proteins in storage cells and the biosynthesis of peptides that occurs during translation at the germination stage [38]. Protein content in individual samples during germination remained at a relatively constant level, which indicates the existence of a balance between degradation and synthesis of protein structures. The significantly higher protein content in radish sprouts developing in white light (3 DAT) may result from the release of larger amounts of proteins from storage material (e.g., albumin or phytates) or proteins associated with the cell membrane, which then underwent proteolysis (4 DAT). This result suggests that the tested lighting variants, except for UV, inhibit enzymes catalysing the hydrolysis of peptide bonds in the seed storage material.

As phosphorus is one of the most important non-metallic elements essential for life, research within “phosphoromics” should focus on phosphorus speciation in plant tissues. ^31^P NMR spectroscopy is a suitable technique for the detailed characterization of all P species, including inorganic and organic P forms, in tested samples of radish sprouts. The type of colour of light used in experiments does not clearly affect the phosphorus profile obtained by the analysis of ^31^P NMR. On the basis of the results obtained, it can be concluded that the tested seeds possess differential phosphorus profiles, although some similarities can also be noted. The ratio of P3 to P4 changed during the course of the experiment. It is worth returning to the influence of UV-30 light, for which the relationships were reversed at 3 DAT, and at 4 DAT the proportion of the P3 form was twice that of the P4 form. Similar relationships were observed for UV-3, but only at 4 DAT. Such a result may indicate the release of orthophosphate ions from the phosphomonoesters as a result of hydrolysis of, for example, sugar derivatives. For the other experimental variants where an increase in the proportion of P4 over P3 was observed, it is most likely that hydrolysis of the ester bonds in the phytic acid molecules took place and the released Pi ions were immediately incorporated into the synthesis processes of the phosphomonoesters.

The results of the experiment proved that the light colour also has an impact on the energy status of plant cells. The analysis of the average coefficients of adenylate content indicates that the light colour exerts the most significant influence on the AMP content. The results obtained for blue, warm white and cold white light are of particular interest, as they had a significant impact on the AMP content on each day of radish growth, with the exception of 2 DAT for warm white light. Furthermore, similarities can be observed in the changes in ADP content during cultivation among the individual tested conditions. In the sprouts treated with red, blue, warm white, or purple light, a regular increase in ADP content was noted on subsequent days of development. In contrast, when exposed to UV light, the values remained relatively constant. The trend of ADP content changes on individual days of the experiment differed from that observed for AMP and ATP. The correlated decrease in AMP and ATP content with an increase in ADP content in the case of blue (2–4 DAT) and purple (3–4 DAT) light may be related to the increased enzymatic activity of adenylate kinase (E.C. 2.7.4.3.), which catalyses the reversible transfer of the γ-phosphate group from ATP to AMP, releasing two ADP molecules. Although some adenylate kinases can catalyse the transphosphorylation of other molecules, the preferred substrate of all isoforms of these enzymes is AMP, and the main phosphate donor is ATP [39]. In addition, plant adenylate kinases are exclusively associated with chloroplasts and mitochondria, and over 90% of the total enzymatic activity is exhibited by those found in mesophyll chloroplasts [40,41,42,43,44,45]. Such alterations in position assist in the avoidance of damage to the chloroplasts that may be caused by the presence of excessive light energy [43]. The analysis of the effect of UV radiation on AMP content demonstrated that these changes were contingent upon the light energy within the 395–400 nm range. The results of the studies indicated that, in the case of UV-3, the AMP content was lower at 2 and 3 DAT compared to the control, while in the case of UV-30, this content was higher. This result suggests that plants developing in UV-30 have an increased energy demand compared to plants developing in UV-3. Furthermore, the observed increase in AMP content from 2 to 3 DAT in plants treated with UV-30, accompanied by a simultaneous decrease in ATP content, suggests the occurrence of hydrolysis of anhydride bonds between phosphorus residues in ATP molecules.

The AEC parameter values in tested radish sprouts treated with different lighting variants indicate that plants subjected to this type of stress maintain a state of homeostasis, with a slight tendency for catabolic processes to prevail. The most probable explanation for this phenomenon is related to the increased demand for the decomposition products of complex compounds, which are reserve substances in seeds. Furthermore, metabolic processes are disrupted in plants that are subjected to stress conditions. To overcome this, alternative biochemical pathways are initiated, whereby the energy initially stored in ATP is continuously depleted. During these transformations, the hydrolysis of high-energy bonds in ATP results in the formation of AMP molecules, which explains the elevated content of this adenine nucleotide in plant cells under non-optimal lighting conditions [46,47,48,49].

In the experiments carried out in the present study, the carotenoid content was significantly lower in samples grown under stress conditions compared to control conditions. The content of carotenoids in radish sprouts treated with UV-30 light at 2 and 4 DAT and red light at 2 and 3 DAT were higher or equal. On the basis of the results discussed, it can be clearly concluded that lighting conditions at the early stage of radish development significantly affect the photosynthetic pigment content and may lead to photosynthetic disorders in developing radish sprouts.

The antioxidant activity is reflected in the synergistic action of a number of antioxidants, including phenolic compounds, ascorbic acid and the cruciferous plant-specific glucosinolates [50]. The study indicates that stressor factor such as lighting also had an impact on the total antioxidant content. The application of UV-3 light has been observed to exert a discernible impact on the antioxidant content, resulting in a notable decline in the concentration of these compounds over the course of their development.

If the total antioxidant content is correlated with the antioxidant activity of radish sprouts, then a significantly lower content of these metabolites (compared to control) indicates their consumption in cellular detoxification processes. The results obtained do not demonstrate a correlation between these parameters. The data indicate that, depending on the variety, the content of phenolic compounds in radish seeds is at the level of 6.1–14.5 mg GAE/g DW [51]. These values are consistent with those previously published in the literature. The impact of light colour on phenolic compound content is also contingent on the plant species [50,52]. The observed effect of red light on phenolic compound content in radish sprouts is consistent with previously obtained results. However, in the case of blue light, the same effect was not observed [53,54]. Fluctuations in flavonoid content can be influenced by environmental signals [55]. Additionally, flavonoids play a role in protecting against UV radiation by filtering light [55,56]. In dicotyledonous plants, which include the radish, flavonoids are found in the seed coat [56]. A review of the literature reveals that flavonoids accumulate transiently in the seedlings and cuttings of a considerable number of plant species [57]. Furthermore, it has been demonstrated that ultraviolet radiation and blue light are responsible for an increase in flavonoid content in buckwheat [58] and *Arabidopsis* sprouts [57]. The dissimilarity between the obtained results and the literature data can be attributed to differences in the control conditions. In the aforementioned examples, the controls were sprouts developing in the dark, rather than in white light. Other studies have demonstrated that UV-B radiation has a significant effect on the biosynthesis of flavonoids, including kaempferol, isoliquiritigenin, apigenin and daidzein, in alfalfa sprouts [59]. However, the aforementioned source data do not corroborate these results. The morphological changes and physicochemical responses of plants resulting from ultraviolet radiation are often contingent upon the intensity of irradiation, the duration of exposure, and the spectral region of UV radiation [60,61]. Ultraviolet radiation can be classified into three distinct categories: UV-A (315–400 nm), UV-B (280–315 nm) and UV-C (100–280 nm) [62]. It is assumed that UV radiation constitutes approximately 5% of the total solar irradiance reaching the Earth’s surface, and that it is devoid of radiation from the UV-C region [62,63]. In sunlight, however, the level of UV-A radiation is 500 to 1000 times greater than that of UV-B. Despite the greater proportion of UV-A, it is regarded as being less harmful than UV-B. The differences between the cited literature data and our own studies can be attributed primarily to differences in exposure time and the range of ultraviolet radiation employed. Nevertheless, the experiments conducted on quinoa appear to be worthy of further investigation [61]. The results of these studies demonstrated that the flavonoid content of leaves exposed to 30 min of UV-B radiation remained unaltered in comparison to the control conditions. Conversely, 60 min of irradiation resulted in a notable decline in the flavonoid content of the leaves. The response of plants to light conditions is likely to be species-dependent, as evidenced by the case of phenolic compounds. Prior research indicates that there are no differences in the content of anthocyanins (a class of flavonoids) in 5-day-old radish sprouts developing in red and blue light compared to the content of these substances in sprouts developing in white light [27]. It is generally accepted that light has a significant effect on the synthesis of secondary metabolites (including phenolic compounds). The results of the experiments indicate that by using different colours of light it is possible to influence not only the content of phenolic compounds but also other antioxidants present in radish sprouts, while increasing their antioxidant potential. However, more in-depth research in this area is needed to analyse in detail the individual compounds with antioxidant activity, e.g., from the glucosinolate group, which are commonly found in radish parts.

Research into the effects of light on seed germination and early plant development has mainly focused on blue, red or ultraviolet light (UV-B, UV-C). Studies have shown that light colour affects not only commonly assessed biochemical parameters, such as antioxidant compounds, but also the metabolism of phosphorus compounds. Light causes changes in the activity of phytases—enzymes crucial to the germination process—and thus affects free phosphorus (Pi) levels. In addition, as shown in the previous sections, depending on the wavelength, light changes the nature of the metabolic processes taking place and affects the energy status of the plant cells.

## 4. Materials and Methods

### 4.1. Plant Material, Seed Germination and Light Treatments

Seeds of radish sprouts (*Raphanus sativus* var. *sativus*) representing the Brassicaceae family were purchased from a local plant breeding and seed company located in east-central Poland (PNOS Sp. z o.o., Ożarów Mazowiecki, Poland) and stored at room temperature in tightly sealed polyethylene bags until use. The untreated seeds were previously sanitized with 70% (*v*/*v*) ethanol for 30 s and 0.5% (*v*/*v*) sodium hypochlorite solution for 15 min, drained and then thoroughly washed with distilled water until they reached a neutral pH. Afterward, 1.0 ± 0.1 g sample of seeds were soaked in distilled water for 4 h in darkness at room temperature, with light agitation. The water-imbibed seeds were placed on two layers of filter paper into Petri dishes (10 cm diameter). The culture was moistened with 5 mL of distilled water twice a day. The samples were cultivated in a plant growth chamber, phytotron FITO DUO (Biogenet, Józefów, Poland) equipped with white (cold white ((CW), 5000 K), warm white ((WW), 2700 K)), blue (blue (B), λ = 460–480 nm; deep blue (DB), λ = 430–450 nm), and red (red (R), λ = 630–650 nm; deep red (DR), λ = 650–670 nm; far red (FR), λ = 710–740 nm), and ultraviolet ((UV), λ = 395–400 nm) LEDs, under dark (24 h) and then under different variants of light conditions in a photoperiod regime with cycles of 16 h light and 8 h darkness at controlled temperature of 25 °C/20 °C (day/night) with 70% relative air humidity. The data on the wavelength of the light sources and light intensity for individual colours of light are presented in Table 2. The experiments were conducted over the five following days (0 DAT was defined after 4 h of soaking). The radish sprouts (in five batches) were taken out of the phytotron every 24 h, starting from 2 DAT. After harvest, the sprouts were immediately frozen in liquid nitrogen, and lyophilized at −50 °C in a freeze dryer ChristAlpha 1–2 LD plus (Osterode am Harz, Germany). The lyophilized samples were ground to a fine powder in a cryogenic mill machine (SPEX 6775 Freezer/Mill; SpexSamplePrep, Metuchen, NJ, USA) and stored at −28 °C until further analysis. The cryogenic mill chills radish sprouts with liquid nitrogen and then pulverises them with a magnetically driven impactor. The milling machine was precooled for 5 min to reach cryogenic temperature before grinding. After that, the samples were milled for 30 s in two grinding cycles at a rate of 15 CPS (cycles per second) with a 1-min intercool.

The reagents used, except malachite green and surfactant (CHAPS), were analytical grade purchased from Avantor Performance Materials Poland S.A. (Gliwice, Poland) and Merck (Merck Millipore, Darmstadt, Germany) and used without further purification. The water was treated in a Milli-Q water purification system (Millipore, Bedford, MA, USA).

### 4.2. Determination of Germination of Radish Seeds

The germination of radish seeds was observed for four days for each tested light condition, after which the number of germinated and non-germinated seeds was counted. A seed was considered to be germinated if its radicle had emerged. The percentage of seed germination (GP) was calculated as follows:

(1) $$\mathrm{GP}=\frac{\mathrm{Number}\;\mathrm{of}\;\mathrm{Germinated}\;\mathrm{Seeds}}{\mathrm{Total}\;\mathrm{Number}\;\mathrm{of}\;\mathrm{Seeds}}\times 100\;[\%]$$

### 4.3. Determination of Acid Phytase Activity and Protein Content in Radish Sprouts

Phytase activity in radish sprouts was measured as described by Kafka et al. [2], with some modifications. A ground radish sample (250 ± 0.5 mg; *n* = 3) was added to ice cold buffer (2 mL of 10 mM Tris-HCl, H 7.0, containing reduced glutathione, 0.5 mM), the suspension was gently shaken, and then solid cetylpyridinium bromide (10 mg, final concentration 0.5% (*w*/*v*)) was added. The suspension was homogenised using a Hielscher UP200HT ultrasonic homogeniser (26 kHz, 200 W, Hielscher Ultrasonics GmbH, Teltow, Germany) for 2 × 30 s with a 15 s delay. The resulting crude homogenate was centrifuged at 13,000× *g* for 30 min at 4 °C. The supernatant containing phytase activity was collected and filtered using a 0.45-µm membrane filter NYLON. Acid phytase activity was determined by measuring the inorganic phosphate (Pi) released from sodium phytate salt by the enzyme. Acid phytase activity was tested in a sodium acetate buffer (100 mM, pH 5.0), containing sodium phytate (1 mM) and CaCl_2_ (1 mM). The assay mixture, including 240 µL of the sodium acetate buffer and 10 µL of extract containing phytase activity was incubated at 37 °C. The reaction was stopped after exactly 60 min by the addition of 50 µL of 50% TCA and the released Pi was quantified by adding 700 µL of ammonium molybdate solution (1:6 solution of 10% *w*/*v* ascorbic acid and 0.42% ammonium molybdate (*w*/*v*) in 0.5 M H_2_SO_4_). The solution was incubated at 37 °C for 10 min, and the precipitated protein was removed by centrifugation at 13,000× *g* for 5 min. Absorbance at 820 nm was measured by UV-VIS spectrophotometer (Rayleigh UV2601 UV/VIS, Beijing, China) against a background sample in which the enzyme was inactivated at the start of the reaction. The Pi concentration was determined from a calibration curve using KH_2_PO_4_ as the standard (absorbance at 820 nm = 2.442 K_2_HPO_4_ (mM) − 0.001, R^2^ = 0.998). One unit of enzyme is defined as the amount of enzyme that releases 1 µmol of Pi from sodium phytate per minute under these conditions. Soluble protein concentration of the supernatant was determined according to the Coomassie blue dye-binding method described by Bradford [64] with bovine serum albumin as a standard.

### 4.4. Isolation of Phosphorus Compounds from Radish Sprouts

A ground radish sample (*n* = 3) of 1.0 ± 0.1 g mass was mixed with 5 mL of 35% (*v*/*v*) perchloric acid (HClO_4_), which was used as an extraction solvent, and sonicated at 25 °C for 30 min using an ultrasonic bath (Cole-Parmer 8891, 42 kHz, 100 W; Vernon Hills, IL, USA). At the end of the sonication process, the whole extract was centrifuged at 5000× *g* for 5 min, and the supernatant was collected. The procedure was conducted three times for each sample. After that, the biomass was washed with 5 mL of distilled water and the suspension was again centrifuged. All collected supernatants were combined, neutralised to pH 7.0 ± 0.5 with potassium carbonate (K_2_CO_3_) and stored at 4 °C for 30 min. Afterwards, the mixture containing insoluble residue and the resulting precipitate of potassium perchlorate (KClO_4_) was again centrifuged. The clear supernatant solution was diluted with deionized water to a final volume of 25 mL and then passed through Whatman No. 41 filter paper. The supernatant was divided into two parts: 2 mL was frozen at −28 °C until the determination of Pi content, and the rest was freeze-dried and stored at −28 °C until ^31^P NMR analysis.

### 4.5. Determination of Inorganic Phosphorus Content (Pi) in Radish Sprouts

The amounts of water soluble inorganic phosphorus forms (Pi) in all extracts of the tested radish sprouts were determined by the spectrophotometric method with the malachite green acid dye procedure, as described by Forlani et al. [65]. Proper sample dilutions in a final volume of 75 µL, obtained with isolation of phosphorus compounds present in the radish sprouts (paragraph 4.5), were supplemented with 1.0 mL of the malachite green–molybdate–acid solution, followed by 0.1 mL of 34% (*w*/*v*) sodium citrate after exactly 1 min. The absorbance of the coloured complex was measured at room temperature at a wavelength of 660 nm against exact blanks for times not longer than 20 min. The amount of orthophosphate ions in the tested radish sprouts was calculated using a calibration curve traced with K_2_HPO_4_ (absorbance at 660 nm = 1.256 K_2_HPO_4_ (mM) + 0.112, R^2^ = 0.996) and expressed as mg of PO_4_^3−^ equivalent per g of dried extract (mg/g dry weight (DW)).

### 4.6. Determination of ^31^P NMR Phosphorus Profiles of Radish Sprouts

The ^31^P NMR spectroscopy analysis was carried out to define the phosphorus profile of the tested radish sprouts. Each freeze-dried extract obtained after radish sprout extraction was redissolved in a mixture of 0.6 mL of deionised water and 0.4 mL of 0.1 M EDTA in 1 M NaOH. Samples were centrifuged at 13,000× *g* for 5 min to remove particles that might contribute to line broadening during NMR analysis. The pH of all the obtained solutions was adjusted to 7.0 ± 0.5. The supernatant (500 µL) was transferred into a 5 mm NMR tube, together with 1mM solution of glufosinate as an internal reference standard (γ = 42.68 ppm). ^31^P NMR experiments were performed using a 400 MHz Bruker Avance DRX spectrometer (Bruker, Rheinstetten, Germany) operating at a 161.98 MHz frequency. Data were acquired at 20 ± 1 °C, using a 30° pulse, a 1.37 s^−1^ acquisition time and a 0.5 s^−1^. relaxation delay. Broadband proton decoupling and a 20 Hz spin rate were used for all samples. The number of scans was 2048. The quantification of P species was performed by spectral deconvolution analysis based on chemical shifts and peak areas. The integration of peak areas was calculated on spectra processed with a line broadening of 2 Hz. ^31^P NMR signal assignments were based on literature data. The relative P concentrations in the neutralised extracts were estimated based on the total NMR signal area and presented as the percentage of each species using TopSpin version 3.6.2 software. All samples were prepared in triplicate for NMR analyses.

### 4.7. HPLC Analysis of ATP, ADP, and AMP in Radish Sprouts

Adenosine triphosphate (ATP), adenosine diphosphate (ADP) and adenosine monophosphate (AMP) were extracted by HClO_4_ solution. The freeze-dried and previously ground sample of radish sprouts (100 ± 0.5 mg) was extracted with 1.0 mL of 6.0 M HClO_4_ and homogenised for 2 × 15 s with a 10 s delay, and the homogenate was centrifugated at 13,000× *g* under 4 °C for 10 min. Then, the supernatant was neutralised in a cold bath with 10.0 M KOH to pH 7.0 ± 0.5 and again centrifuged. The supernatant was collected and diluted to 1.0 mL and stored under −28 °C. Before analysis, the solution was passed through a 0.22-µm membrane filter NYLON.

The content of adenylates in germinated radish sprouts was assessed by reversed phase high-performance liquid chromatography (HPLC) using a Dionex Ultimate 3000 HPLC systemequipped with diode array detector (DAD-3000RS) (Thermo Fischer Scientific, Waltham, MA, USA). The mobile phase consisted of two solvents: A—0.05 M phosphate buffer pH 7.0 with 1% of acetonitrile (ACN); B—ACN. A 30 min gradient elution with variable flow rate was used with the following conditions: 0–10 min 0/100%, A/B, 0.5 mL/min; 10–15 min 25/75%, A/B, 1.0 mL/min; 15–20 min 25/75%, A/B, 1.0 mL/min; 20–25 min 0/100%, A/B, 0.5 mL/min; 25–30 min 0/100%, A/B, 0.5 mL/min. A total of 20 μL of each prepared sample, maintained in an autosampler at a temperature of 8 °C, was injected into a Gemini NX-C18 column (250 × 4.6 mm, Phenomenex Part Number: 00G-4454-E0), equipped with a dedicated SecurityGuard ULTRA Cartridge System, which were placed in a thermostat at a set temperature of 30 °C, with the retention times and peak intensities of separated compounds assessed at 254 nm. The external standards of ATP, ADP, and AMP (6.25–500 µM) were applied to quantify the adenylate contents and expressed as µg/g DW. The adenylate energy charge was determined as follows:

AEC = [(ATP) + 0.5(ADP)]/[(ATP) + (ADP) + (AMP)](2)

### 4.8. Determination of Photosynthetic Pigments (Chlorophylls and Carotenoids) in Radish Sprouts

The contents of photosynthetic pigments (chlorophylls and carotenoids) were determined by the method of Złotek et al. [66] with some modifications. A total of 50 mg of homogenized radish samples was taken in a test tube and 25 mg of MgO was added to prevent the formation of pheophytin. A quantity of 2 mL of 80% (*v*/*v*) acetone solution was added to each sample and homogenized on a platform shaker at 600 rotations per minute (rpm) (Heidolph Vibramax 100; Schwabach, Germany) for 2 h in darkness. The sample was then centrifuged at 13,000× *g* for 5 min at 4 °C. The absorbances of the collected supernatants were measured using 80% (*v*/*v*) acetone as a blank at three different wavelengths: 470, 645, and 663 nm. Concentrations of total chlorophyll and carotenoids were calculated by the following equations:

chlorophyll = chlorophyll a + chlorophyll b = [(12.72 A_663_ − 2.59A_645_) + (22.88A_645_ − 4.67A_663_)] (3)

carotenoid = (1000A_470_ − 3.27 × chlorophyll a − 104 × chlorophyll b)/229 (4)

The results were expressed as mg/g DW.

### 4.9. Determination of Antioxidant Capacities and the Content of Antioxidants, Phenolic Compounds and Flavonoids in Radish Sprouts

#### 4.9.1. Preparation of Aqueous Extracts from the Radish Sprouts

A quantity of 100 ± 0.5 mg of ground radish sample was extracted with distilled water (final volume 2 mL), during 1 h, under stirring on a platform shaker at 600 rpm and light protection. Then, the extraction was placed in an ultrasonic bath at room temperature for 20 min. Finally, the extracts were filtered through a 0.45-µm membrane filter NYLON, and the solutions were stored under −28 °C until further analyses

#### 4.9.2. Antioxidant Capacities of Radish Sprouts

Antioxidant capacities were assayed by DPPH methods. For DPPH free radical-scavenging activity (RSA) assay, 2 mg of DPPH (1,1-diphenyl-2-picrylhydrazyl) was dissolved in 50 mL of methanol and kept for 30 min under dark conditions before testing absorbance at 517 nm. A quantity of 50 µL of each radish extract (*n* = 3) was added to 1.95 mL of DPPH solution in 2 mL Eppendorf tubes. Due to the colouration of the extract, it was necessary to prepare a background blank, which consisted of 50 µL of each radish extract added to 1.95 mL of methanol (without DPPH). The tubes were left in darkness for 30 min at room temperature, and the absorbance was measured at 517 nm using a spectrophotometer. Methanol was used to set the spectrophotometric zero. The RSA of the sample is expressed as the percentage discolouration of the DPPH solution using the following equation:

(5) $$\mathrm{RSA}\;(\%)=\frac{A_{\mathrm{blank}}-{(A}_{\mathrm{sample}}-A_{\mathrm{background}})}{A_{\mathrm{blank}}}\times 100\%$$

#### 4.9.3. Total Antioxidant Compound Content in Radish Sprouts

The total content of antioxidant compounds was determined according to Korzeniowska et al. [67], with minor modifications. The ABTS working solution was used with Trolox as a positive control. The ABTS working solution was prepared by mixing 1 mL of ABTS (2.2’-azino-bis (3-ethylbenzothiazoline-6-sulphonic acid)) solution (14 mM) with 1 mL of a potassium persulfate solution (5 mM) and incubated overnight under dark conditions. After that, the mixture was appropriately diluted with water to obtain an absorbance of 0.750 ± 0.050 at 734 nm. Up to 100 µL of aqueous extracts from the radish sprouts (*n* = 3), 1 mL of ABTS working solution was added, and the absorbance was measured after 6 min at 734 nm against water. The absorbance of the extract without reagent was measured as the background of the sample. The total antioxidant content was calculated from the standard curve for methanolic Trolox solution (absorbance at 734 nm = −0.011 Trolox (μg/mL) + 0.705, R^2^ = 0.999)), and the results were expressed as mg Trolox/g DW.

#### 4.9.4. Total Phenolic Assay of Radish Sprouts

The total phenolic content (TPC) of the extracts was determined according to Vale et al. [68] with minor modifications. The Folin–Ciocalteau reagent was used with gallic acid (GA) as positive control. A quantity of 50 μL of the aqueous extracts (*n* = 3) from the radish sprouts was mixed with 2.5 mL of Folin–Ciocalteau reagent (diluted 1/10) in a 10 mL screw-cap tube. After adding 2 mL of Na_2_CO_3_ (7.5%, *w*/*v*) the tube was closed and kept at 45 °C for 15 min. The absorbance of all samples was measured at 765 nm. The TPC was calculated using a calibration curve traced with GA (absorbance at 765 nm = 0.001 GA (μg/mL) − 0.074, R2 = 0.998)) and expressed as mg GA equivalent per g of DW (mg GAE/g DW).

#### 4.9.5. Total Flavonoid Assay of Radish Sprouts

The total flavonoids content (TFC) was determined with aluminium chloride (AlCl_3_) according to Zhishen et al. [69] using quercetin (Q) as positive control. Briefly, 100 μL of the aqueous extracts (*n* = 3) from the radish sprouts was added to 300 μL of distilled water followed by 30 μL of NaNO_2_ (5%). After 5 min at 25 °C, 30 μL of AlCl_3_ (10%) was added and the solution was allowed to stand for a further 5 min. Then, the reaction mixture was treated with 200 μL of NaOH (1 mM) and topped up to a volume of 1 mL with distilled water. The absorbance of the mixture was then determined at 510 nm against a water blank. The flavonoids content was calculated from a Q standard curve (absorbance at 510 nm = 0.001 Q (μg/mL) + 0.002, R^2^ = 0.999). Results were expressed as mg Q/g DW.

### 4.10. Statistical Analysis

All experiments were carried out in at least three independent replicates, and data are expressed as the mean ± standard deviation. Statistical and graphic analyses were performed using OriginPro, Version 2021b software (OriginLab Corporation, Northampton, MA, USA). Data were analysed with one-way ANOVA and tested for significant (*p* ≤ 0.05) differences using Tukey’s test.

## 5. Conclusions

So far, only the standard set of parameters has commonly been used for qualitative and quantitative assessment of plant condition. The principal criteria for assessing seed quality and condition are germination strength and energy. The results concerning the effect of light on radish seed germination, as a model plant, clearly show that the use of only commonly accepted parameters characterising plant health, such as germination energy and growth profile, does not provide meaningful information on the effect of light colour on the germination process and radish sprout development. Meanwhile, the physiological status of plants may be much better characterised based on chosen chemical parameters of their development and growth. Based on the results of our study, we may propose the phosphoromic approach as an important metabolic parameter. We observed that seeds, as well as plants at an early stage of development, show high resistance to emerging abiotic stresses, which may indicate the existence of mechanisms to maintain homeostasis.

## Figures and Tables

**Table 1:** Relative contents (%) of labelled forms of phosphorus in neutralised extracts of radish sprouts developing under different light conditions.

DAT	Light Version	P1 (%)	P2 (%)	P3 (%)	P4 (%)
2	White (Control)	–	0.7	40.5	58.8
	Red	–	1.4	40.8	57.8
	Blue	–	1.1	40.1	58.8
	Warm white	–	1.0	41.9	57.1
	Purple	–	1.5	37.1	61.4
	Cold white	–	1.1	31.7	67.2
	UV-3	–	3.8	47.6	48.6
	UV-30	–	1.2	54.4	44.4
3	White (Control)	–	1.3	51.5	47.2
	Red	–	0.6	41.9	57.5
	Blue	–	0.6	37.1	62.3
	Warm white	–	0.7	37.2	62.1
	Purple	–	0.8	36.5	62.7
	Cold white	–	1.2	33.4	65.4
	UV-3	–	2.0	49.9	48.1
	UV-30	–	4.9	40.7	54.4
4	White (Control)	–	1.7	27.5	70.8
	Red	–	1.8	30.0	68.2
	Blue	–	0.4	34.6	65.0
	Warm white	–	0.6	35.3	64.1
	Purple	–	0.5	33.6	65.9
	Cold white	–	0.8	27.1	72.1
	UV-3	–	1.3	69.0	29.7
	UV-30	–	1.7	62.4	35.9

The range of chemical shifts (ppm) of the individual phosphorus forms recorded on the ^31^P NMR spectra of the neutralised extract of the test samples: P1—polyphosphates/diphosphates ((−15)−(−3.5) ppm); P2—phosphodiesters ((−1)−1.5 ppm); P3—phytates/orthophosphates (1.5−3.7 ppm); P4—other monoesters (3.7−6 ppm); ‘−’—not indicated.

**Table 2:** Different variants of light used during the germination of radish sprouts. The share of individual sources of light is presented in percentages.

Sources of Light	Colour of Light							
	White (Control)	Blue	Red	Warm White	Purple	Cold White	UV-3	UV-30
Cold White (CW) [%]; 5000 K	50					100	50	50
Warm White (WW) [%]; 2700 K	50			100			50	50
Blue (B) [%]; λ = 460–480 nm	50	100			100		50	50
Deep Blue (DB) [%]; λ = 430–450 nm	50	100			100		50	50
Red (R) [%]; λ = 630–650 nm	50		100		100		50	50
Deep Red (DR) [%]; λ = 650–670 nm	50		100		100		50	50
Far Red (FR) [%]; λ = 710–740 nm	50						50	50
Ultraviolet (UV) [%]; λ = 395–400 nm							3	30
Light intensity [kLux]	5.05 ± 0.05	3.54 ± 0.01	4.61 ± 0.01	4.15 ± 0.02	4.10 ± 0.2	4.9 ± 0.2	5.76 ± 0.2	6.22 ± 0.2

## Data Availability

On request to those interested.
